# A Phenomenological Study on Patients' Experiences With Extraction Retraction Orthodontic Regret

**DOI:** 10.7759/cureus.80728

**Published:** 2025-03-17

**Authors:** Jeremy Antepyan-Ruckenstein

**Affiliations:** 1 Pharmacy, Independent Researcher, Coquitlam, CAN

**Keywords:** craniofacial abnormalities, facial pain, orthodontics, orthodontic space closure, post-traumatic, psychological trauma, sleep apnea syndromes, stress disorders, temporomandibular joint disorders, tooth extraction

## Abstract

Background

Extraction retraction orthodontic (ERO) practices are commonly used to treat every malocclusion. Occasionally, patients express dissatisfaction over the previous ERO treatment. This study investigates the experience of those who have had ERO intervention and expressed regret or dissatisfaction with this treatment.

Methods

Semi-structured interviews were conducted with patients who have expressed regret over past ERO treatment. Interpretive phenomenological analysis (IPA) was used to derive themes from transcripts.

Results

Eleven participants were recruited, who gave informed consent and participated in the semi-structured interview process. Six major themes were identified through IPA: “ERO Treatment Course”, “Lack of Informed Consent”, “Ocean of Grief and Trauma” to describe the overall patient emotional experience, “Multifaceted Health Complaints”, “Finding Solutions and Coping Strategies”, and “Wishing There Was a Better Way”. Participants felt like they were not able to give informed consent for ERO due to a number of different reasons such as being too young, not being given accurate information on the risks, or being influenced parentally, culturally, or by the provider. Participants regret ERO due to a number of multifaceted health complaints, including but not limited to sleep breathing disorders, craniofacial pain patterns, neuropsychobehavioural symptoms, and negative aesthetic outcomes that they believe result from ERO.

Conclusion

Patient regret following extraction retraction orthodontics is due to a lack of informed consent and negative health and aesthetic effects. To prevent this regret, future research and practice direction should focus on improving the informed consent process, promoting optimal dentofacial development and non-extraction-retraction therapies.

## Introduction

The incidence of malocclusion and dentofacial deformity (DFD) is high, with reported rates varying between 39-93% in some morphologies [1-3]. Despite this great need, there is little consensus on treatment, with multiple systematic reviews generally finding low-quality evidence in various interventions [4-7]. Understanding the multifaceted etiology and patient-centered, evidence-based treatment protocols are needed to prevent the development and treat the occurrence of DFD and related sequelae.

Extraction retraction orthodontic (ERO) practices include the extraction of healthy teeth for orthodontic purposes and/or retractive forces applied on teeth or craniofacial structure [8,9]. Orthodontic extractions with retraction of teeth are performed frequently in practice for every class of malocclusion with reported rates varying between 34.4-89% [10-13]. A retractive headgear uses an externally anchored device to create a retractive force on teeth or jaws for the treatment of prominent front teeth and/or anteroposterior skeletal growth discrepancies and is often used in practice today [14-16].

Extraction retraction regret is colloquially used to describe regret with prior ERO treatment [9]. Patients may present with any number of com­­plaints, including sleep-disordered breathing, jaw joint or craniofacial pain patterns, or aesthetic complaints such as a long, retruded face and attribute this to ERO. Numerous studies have been conducted examining the link between ERO and these complaints, and no definitive link has been established, but the quality of research is low, the risk of bias high, and the phenomenon continues [17-20]. 

There have been studies examining patient experience with ERO [21,22] but little phenomenological research and none looking specifically at dissatisfaction. As such, this study aims to understand the subjectivity of experiences of patients who have expressed regret or dissatisfaction with ERO. The study aims to explore the etiology of this regret, what complaints these patients attribute to the treatment, and their experience seeking aid. The results we obtain should be useful for future research and practice innovations.

## Materials and methods

A qualitative research methodology was used. This study adheres to the COnsolidated criteria for REporting Qualitative research (COREQ)-32 criteria for reporting qualitative research (Appendix). Ethical approval was obtained from the Canadian Shield Ethics Review Board (approval number 2022-12-001). 

Design

A phenomenological approach was chosen for this project as it aims to explore the lived experience of patients with regret or dissatisfaction following ERO treatment. Phenomenology as a qualitative research method is concerned with meaning, how individuals experience the world and create understanding of events, to elucidate the nature of the reality that holds the phenomenon [23]. Through exploring the subjective experience of those who regret prior ERO treatment in a double hermeneutic thematic interpretivist fashion, novel understanding can be obtained regarding the etiology of this dissatisfaction that may guide future research and care.

Inclusion and recruitment

A purposive convenience sample of adult English-speaking patients who have had ERO treatment and expressed regret or dissatisfaction over such was recruited. Recruitment was done through online messaging boards, social media groups, and healthcare providers’ practices. Written informed consent was obtained from all participants. As the interpretive phenomenological analysis (IPA) calls for smaller homogeneous sample sizes to allow for richer analysis, a planned sample size of five to 15 was established, and recruitment would continue until data saturation was achieved. Individuals who did not undergo premolar extraction with space closure or retractive headgear treatment were excluded from the study.

Data collection

Interviews were conducted via video-conference using Zoom software and a predefined semi-structured questionnaire (Table 1). Questions considered the dimensions of overall experience, nature and onset of regret, affected aspects, and experience seeking aid. Interviews were audio-recorded and transcribed verbatim. Data saturation was achieved when, upon iterative reflection, participants described a shared thematic ERO treatment experience similar to that of previous participants.

**Table 1:** Interview guide

Opening	To begin the interview, I would like to remind you to share as much as you feel comfortable with and to take breaks at any time during the interview. This isn’t a test or an interrogation, let me know if there’s anything you need to feel comfortable
Experience with extraction-retraction orthodontics	To start off, can you tell me about your past experience with extraction-retraction orthodontics?
	When did you receive this treatment? At what age or year?
	What exactly was done? Which teeth were extracted, how were teeth moved, where was headgear and force applied?
	Why did you receive this treatment?
	What did you understand about this treatment at the time?
	Did you want this treatment?
	Were there positive aspects to this treatment?
	Why, if applicable, did you begin to regret this treatment?
	Has your understanding of this treatment changed since you received it?
	Do you think your experience with this treatment is similar to other peoples?
Affected aspects	How has your past extraction-retraction orthodontic treatment affected you?
	Has extraction-retraction orthodontic treatment affected your physical health?
	As above with mental health?
	Can you describe what emotions come up for you in regards to your past extraction-retraction orthodontics?
	As above with physical appearance?
	As above with social, professional, experiences?
Experience seeking aid	What have you tried to improve your condition?
	What have you found that benefitted your condition?
	Were you able to find help?
	What has been your experience with healthcare providers while seeking aid?
Closing	Is there anything else related to your prior extraction-retraction orthodontic treatment you would like to add?
	Is there anything you would like to ask?
	Would you like to review the transcript of your interview to possibly correct or add anything?

Analysis

Participants were given pseudonyms to de-identify the interviews. Interview transcripts were reviewed extensively for familiarization, and summary descriptive and conceptual notes were added line-by-line. A two-phase constructivist approach to analysis was undertaken by the researcher. In the first phase, intrapatient thematic codes were deduced constructively and iteratively as described by Smith et al. (2009) through reflection on the annotated comments and grouping of similar concepts [24]. Intrapatient themes were reviewed for familiarity, and interpatient themes were constructed iteratively through ongoing reflection in a similar manner to the first phase. The manuscript and annotated transcripts with superordinate themes were submitted to participants for feedback before publication to ensure accuracy and ongoing informed consent.

## Results

Eleven participants who gave informed consent were recruited and participated in the semi-structured interview process between April and May 2023. Interviews lasted 23-97 minutes (mean 54), varying in duration depending on patient verbosity and narrative depth. Participants varied in demographic information, original ERO intervention, health complaints, and therapies sought to help (Table 2).

**Table 2:** Participant Characteristics ERO: extraction retraction orthodontic. ADHD: attention deficit hyperactivity disorder. ALF: advanced lightwire functional appliance. CBD: cannabidiol. CPAP: continuous positive airway pressure therapy. GERD: gastroesophageal reflux disease. EASE: endoscopically assisted nasomaxillary expansion. MAD: mandibular advancement device. MMA: maxillomandibular advancement surgery. OSA: obstructive sleep apnea. SFOT: surgically facilitated orthodontic treatment. TMJ/D: temporomandibular joint disorder. URTI: upper respiratory tract infection. Participants were given pseudonyms to de-identify the interviews.

Alias	Age	Gender Assigned at Birth/Pronouns	Place of Birth/Residence	Highest Level of Education	Occupational Field	ERO Intervention; indication; age received	Multifacetted Health Complaints	Therapies Sought to Help or Reverse Damage
Alice	30	female, she/her	China/Australia	Undergraduate	Healthcare	Four premolar extraction retraction; prominent upper front teeth; roughly twenty years old	Poor facial aesthetic results (recessed, longer face, premature aging), severe TMJ and craniofacial pain, negative social, professional, emotional consequences, poor sleep quality, difficulty breathing, difficulty maintaining good lingual palatal posture	Orthodontic reopening premolar extraction sites for implants
Jordan	28	Male, he/him	US, France	Graduate	Physical education	Two upper premolar extraction retraction; crowding, slight underbite; childhood	Chronic bronchitis, ADHD, looking tired, retrognathic, diagnosed OSA, poor aesthetic results, severe somnolence, negative social/professional/academic impact, long face, post nasal drip, URTI, purple lines under eyes, always sleep deprived, poor exercise recovery, lower back pain, difficulty nasal breathing, forward head posture, kyphosis, negative emotional mental health, lack of impulse control, hypomania, anxiety, reduced ability to read and respond to social cues appropriately, brain fog, Asperger's syndrome,	MMA, CPAP, MAD, antidepressants, stimulant medications, nasal surgery, asthma inhalers, anxiolytics,
Kalil	35	male, he/him	India, US	Undergraduate	Software production	Four premolar extraction retraction, wisdom tooth extraction; severe crowding; childhood	Retrognathia, craniofacial recession, sleep apnea, tinnitus, malocclusion, asymmetry, eustachian tube dysfunction, difficulty breathing, TMJD, ADHD, vertigo, migraines, negative psychosocial health, low cardiovascular capacity	Nasal dilators, ALF orthodontic appliance, EASE nasomaxillary expansion, oral myofunctional therapy, exercise, mouth tape, CPAP, face pulling,
Nancy	45	Female, she/her	China	College	Model agency	Four tooth extractions retraction, three bicuspids, one canine, three wisdom tooth extraction; edge to edge bite, crowding; Forty one years old	Lack of tongue space, nightmares, difficulty smiling, sleep disruption, chewing difficulty, different malocclusion, shortness of breath on exertion, fatigue, malaise, heartburn, formication, difficulty concentrating, low mood, rumination, negative emotional/professional/social effects	Medications, exercise, diet modification
Jessica	45	Female, she/her	Scotland, US	Undergraduate	Social media and advocacy	Two upper premolar extraction retraction, wisdom tooth extraction; crowding, impacted canines; twelve years old	OSA, poor sleep quality, low functionality, depression, anxiety, ongoing malocclusion, myofacial tension, forward head posture, social isolation, heart palpitations, long narrow recessed face, negative impact on social and professional life, brain fog	Failed tooth born adult palatal expansion, lingual frenectomy, massage therapy, CPAP, patient advocacy, healthy diet, antidepressants, psychotherapy, meditation, yoga, myofunctional therapy,
Nick	22	Male, he/him/his	US	Undergraduate	Horticulture	Four premolar extraction, retractive headgear, four third molar extraction; overbite, crowding; around seven years old	Nasal obstruction, difficulty maintaining good oral posture, difficulty breathing, retromicrognathia, difficult with lip closure, smaller airways, poor sleep quality, slight open bite, forward head posture, kyphosis, limited exercise potential, insecurity about physical appearance, low self confidence, intermittent depression, attention deficit, negative social impact	Nasal surgery, exercise, antidepressant,
Henrietta	64	Female, she/her	Canada	Graduate	Healthcare	Four premolar extraction retraction, wisdom tooth extraction; prominent upper front teeth, crowding, impacted front teeth; ten years old	Difficulty swallowing, relapse to poor occlusion, snoring, poor sleep quality, diagnosed OSA, GERD, poor oral health resulting in abscess and further extractions, lack of tongue space, hypertension, anxiety, depression, negative professional impact	Diet, sleep behavioural modification, exercise
Laura	59	Female, she/her	US, France	PHD	Professor	Four premolar extraction retraction; no initial discernable malocclusion; fourteen years old	negative emotional/social/financial effect, low functionality, negative aesthetic outcome (retrusion), asthma, fatigue, forward head posture, negative effect on athletic performance, breathing difficulty, nightmares, poor sleep, lack of tongue space, TMD, tinnitus, swallowing difficulty, dizziness, vertigo, bruxism, bone loss in face, depression, miscarriages, lip biting and lip closure difficulty, smoking cigarettes, drinking alcohol	Botox, transverse maxillary expansion, psychotherapy, advocacy, peer support, orthodontic expansion, partially orthodontically reopening premolar extraction sites, SFOT, oral myofunctional therapy, opioid analgesia, CBD, faith
Paula	36	Female, she/her	US	Undergraduate	Health coaching	Four upper premolar extraction retraction, retractive headgear; severe crowding, crossbite, impacted canine; a few years older than seven	Poor aesthetic results (long, narrow, retruded face), ongoing malocclusion, deviated septum, poor nasal breathing, chronic fatigue, poor sleep quality, hormonal irregularities, head and neck pain, hypothyroidism, amenorrhea, negative emotional/financial/social/professional effect	septoplasty, MMA, lingual frenectomy, naturopathy, hormone replacement, psychotherapy, dietary interventions, exercise therapy, various diagnostic tests, various complementary alternative medicine interventions, medications
Rick	44	Male, he/him	Canada	Graduate	Property Manager	Retractive cervical headgear; no discernable malocclusion; eleven years old	Fatigue, low functionality and unable to perform activities of daily living, obstructive sleep apnea, retrusive facial features, forward head posture, neck head and back pain, TMJ problems, negative emotional, financial, professional, social, familial, romantic consequences	CPAP, mandibular advancement device, supplements, complementary alternative medicine
Warren	33	Male, he/him	Sweden	Trade school	Disability pension	Four premolar extraction retraction; prominent upper front teeth; eleven years old	Fatigue, low functionality, poor sleep quality, obstructive sleep apnea, lack of tongue space, severe back pain and injury, head and neck pain, poor posture, poor mobility and physical health, poor craniofacial aesthetic results, negative social/emotional/financial/professional effects	Medications

**Figure 1:**
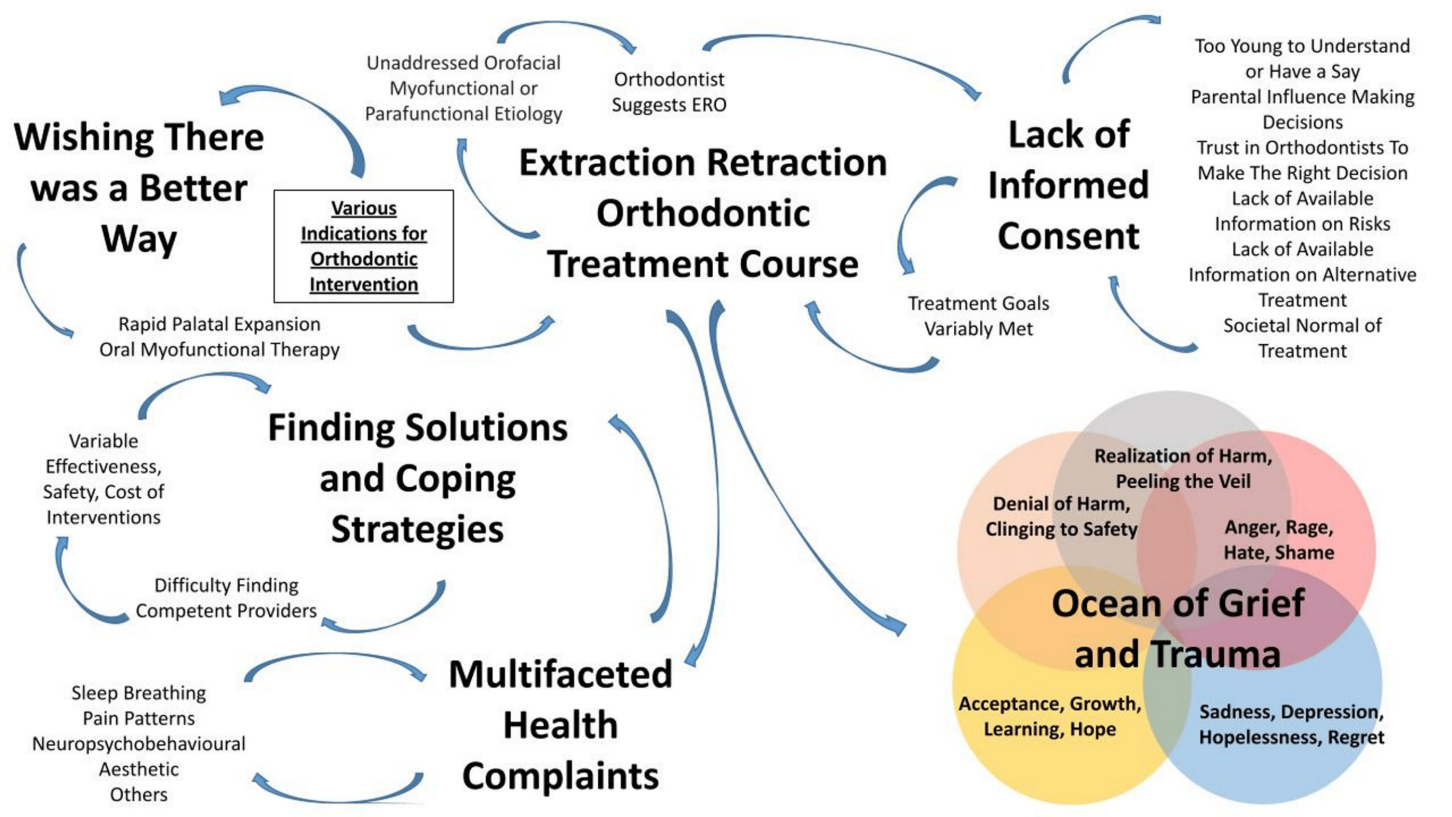
Interactions between interpatient themes

All participants underwent ERO in the past and had health problems that they attributed to this treatment. The analysis identified six major interpatient themes: "ERO Treatment Course", "Lack of Informed Consent", "Ocean of Grief and Trauma", "Multifaceted Health Complaints", "Finding Solutions and Coping Strategies", and "Wishing There Was a Better Way". These themes are expanded upon below, detailed in Table 3, and illustrated in Figure 1.

**Table 3:** Interpatient themes and supporting quotes ERO: extraction retraction orthodontic. ADHD: attention deficit hyperactivity disorder. MHC: multifaceted health complaints. MSE: maxillary skeletal expander. TMJ/D: temporomandibular joint disorder. Participants were given pseudonyms to de-identify the interviews.

Theme	Subthemes	Examples
ERO Treatment Course	Various Motivations for Seeking Orthodontic Intervention	Rick: I didn't have malocclusion. I was minorly bucktoothed.
		Nick: I knew my teeth were crooked. I knew my mouth was crowded
		Warren: I was diagnosed with overbite
		Alice: Originally, my front two incisors, the first two teeth, they are a bit inclined too much.
		Henrietta: I wanted straight teeth, yeah. It was like people would make fun of me... They call me names because I had really bad cosmetic it looked really quite significant.
		Paula: I had one of my canine teeth was on the roof of my mouth...
		Laura: [my siblings and I] all have straight teeth. there's class one occlusion, straight teeth, slight mandibular retrusion, but so slight that it's just attractive. I mean, no breathing issues, no nothing.
		Nick: I had an overbite, crooked teeth
		Jessica: Canines were really high up... the dentist was just like, you have to go to the Orthodontist because your teeth are coming in all crazy.
		Nancy: I never have the plan for the, um, I don't know how to say, the treatment. I just have, like, one teeth have some problems. I went to see the dentist introduced by one of my friend. So I totally go there, like, five times. The first time he saw me, he's doing the cure for my number 6 teeth. He said I have problem for the biting problem. He said that I have the, I don't know how to say. Maybe I can find one picture to show you. [It was an edge to edge bite]
		Jordan: Crooked teeth... I have underbite, and now I wouldn't know specifically
	Unaddressed Orofacial Myofunctional or Parafunctional etiology	Nick: I don't remember anyone ever talking to me about tongue posture, or nose breathing. I think it was always just kind of accepted like I just did it. And I don't think anyone ever really questioned it or even noticed.
		Rick: Well, breastfeeding is really important... Why are we eating soft foods? Because fucking we're stupid capitalists
		Henrietta: I was a thumb sucker... I was told that I wasn't swallowing properly
		Paula: I was mouth breathing... I also sucked my thumb as a kid... I was still tongue tied... I had a lot of allergies
		Warren: The reason I got this, I think, is because I suck my thumb, like, too much and too long as a kid. And I also used the pacifier too much.
		Jessica: Yeah, I was a thumbsucker, mouth breather, had terrible allergies. I had asthma as a child... I had my tongue tie released
	Orthodontist Suggests ERO	Henrietta: of course it's always the orthodontist guy who suggests, okay, you need to go prior to get your teeth extracted so we would have room to make adjustments
		Rick: he says, so your upper jaw is overgrown. Your upper jaw is overgrown, and we need to pull it back to let your lower jaw catch up...
		Alice: she then told me that my oral space, like my arch or my space somewhere there is too small. It's not enough for her to actually push the teeth back. So she said that therefore, we need that space, so we need to take the teeth out
		Warren: And then all of a sudden, they said, yeah, now we're going to pull some teeth
		Kalil: they said that it's either surgery or we have to extract these four teeth to straighten the teeth out
		Paula: they basically decided they had to pull teeth
		Laura: I go in and the orthodontist says, agrees that my occlusion is fine, but says, oh, no, she's got four impacted first premolars. We've got to take them out.
		Nancy: She said I need to do the treatment [pull four teeth/ERO] for to make it like this [good occlusion]
	Treatment goals variably met	Henrietta: [I was satisfied with the treatment at the time]. Looked cosmetically good. [All the teeth were straight]... I wasn't made fun of [after the treatment]
		Jordan: well, maybe the whole sleep apnea thing was developed because I got premolar extractions, which led to receding jaws, unattractive face and potentially reducing the size of the airway
		Rick: Then I went to [a university]. I read everything I could on headgear, all said the same thing. Doesn't make your face grow, makes the face move backward. Backward growth of the face.
		Warren: that's probably the only positive. My bite is still good. I have good, I think it's called occlusion, all the way around.
		Paula: I remember when I got them off kind of like looking in the mirror and being like, oh, that's not really what I expected the outcome to be because it kind of looks like everything was sort of like pulled together....
		Laura: Jaw structure was beautiful before. It slants inward. The neck is going forward. Suddenly I'm diagnosed with asthma... [my mother] said, [name redacted] was such a beautiful girl. Look how ugly is she is now... I have no chin. I'm so retruded... No, there was nothing positive about it... So after he took them off, my mother yelled at him for making me ugly. And he put them back on. So I had reversal age 14 to 18.
		Alice: when I had my braces off, I said, but what about this gap? It's still there. It's quite visible. And that orthodontist said to me that when you wear your retainer, it's going to close itself... Yes, it was straight up, and then my bottom teeth were quite crowded, and then my bottom teeth was straightened up, so that was good. But I think that was all that I would say I'm happy with, to be honest. Crowded teeth, it's actually not a problem for me...
		Nick: I think they [the treatment goals] were definitely met. I don't know if you can see, my teeth are still really straight. I don't think I've had any significant movement. I wear my retainer. My bite, I think is fine. My teeth align well. I don't have any problems chewing, no significant jaw pain, nothing like that. For me, I think it's more about the long term negative consequences that I've started to look into and understand now that I'm kind of seeing, like, maybe that was a little hastily done, but at the time I was satisfied with the straightening and the bite alignment and everything.
		Jessica: I mean, my teeth look beautiful now. I know. I think at the time, I knew nothing about this stuff. Right. So I had a really high arch palate and super narrow, but the teeth that you could see were super straight, and I was quite pleased with how it looked, and it was way better than it was before, with all my canines coming in all crazy and okay.
		Nancy: Even my bite relationship is not so good because I pull like three number four and one number six. So means it cannot be correct. She didn't do this well.
		Kalil: And for what it's worth, he did straighten the remaining teeth I had left, but in doing so damaged my entire facial form.
Lack of Informed Consent	Too Young to Understand or Have a Say	Henrietta: I felt really bad about it. But at that age you just go about what people tell you to do
		Paula: I was a child, I didn't know to question any of that
		Laura: Did I want the treatment? I didn't understand anything about the treatment. I didn't understand why I needed them. I didn't understand any of it... I'm like, you can't regret something that you have no choice in.
		Nick: Well, I think to me, it was not super clear. I was young, I knew my teeth were crooked. I knew my mouth was crowded. So for me, it was just, we're going to improve your smile so your teeth will get straight, your bite will be fixed, and you'll be set. I don't know how much more you can really explain to a young child.
	Parental Influence Making Decisions	Kalil: First of all, it wasn't really my decision, it was my parents
		Henrietta: Just such a young age, you go by what your parents tell you
	Trust in Orthodontists To Make The Right Decision	Warren: But at that age, you don't really understand everything or you trust every adult, and especially if it's a profession like a dentist or in this case, orthodontist I really didn't want to do it at first, but I didn't really have a choice
		Alice: I think, well, you are the professional one, right? I'm not. Of course, I believe you. I think that's basically my thought process. And I didn't actually didn't question because she said... And I feel like at that time there's not much like information out there
		Paula: I think it was: you're trusting the orthodontists to be orthodontists, to be the experts
		Jessica: So it wasn't like they asked, how do you feel about us extracting these teeth? They just were like, this is what we just need to do. So I didn't really question it. And when they pulled my teeth out, it wasn't super fun, but I was kind of like, I guess this is what people do.
		Kalil: they did the best that they knew what they could do, which is to go to an Orthodontist who you should be able to trust that would know the right treatment
	Lack of Available Information on Risks	Rick: So he said, there are no side effects. It's perfectly safe... he says, there's one side effect, your lower jaw could overgrow
		Nancy: I do this search similar, like Google, on the Internet, I saw you shouldn’t do, I don't know how to say, you shouldn’t do, like, [have an edge to edge bite] make this [have a normal occlusion]
		Jordan: He didn't explain what he wanted to do or why it was necessary to extract the teeth. I was 14, and I really didn't look into it. I was just like, oh, we got to extract your teeth to make them straight. I was like, okay. And my mom wasn't too involved either. So we just followed the medical advice.
	Lack of Available Information on Alternative Treatment	Nancy: No, if you need to pull four teeth, I don't want to do this
		Kalil: My parents didn't really want to do the extraction, but they felt they had no choice, and doctors saying, this is what you have to do
	Societal Normal of Treatment	Nick: it's hard for me to remember because I was young, what's the justification for doing all this stuff? But I think it was probably just like the norm
		Paula: I think also at the time maybe it's still is common. So you think it's normal if it's common.
		Henrietta: I think at that time, that was quite common to have the teeth out
		Laura: Age twelve, everyone is getting braces. It's cool. 1970s, it was cool. And, and like hot for middle class, it was a status symbol. And I was excited when one day my mother said, hey, let's take you to the orthodontist to see if you need braces... I wanted braces because I thought it was cool.
		Nick: I think it was probably just like the norm, I should say, to do orthodontics and get started at a young age.
		Nancy: pull four number teeth is normal.
Ocean of Grief and Trauma	Denial of Harm, Clinging to Safety	Kalil: when I first discovered all this and made the connection, there was first denial. It's like, oh, I didn't get affected that bad. I went and measured my intermolar width and try to get a sense of my palate, and I was like, oh, it's not that bad
		Jordan: until recently, I was never aware that teeth extraction had the potential to modify your facial and cranial shape
		Paula: at the time, there was no connection for me between having had the teeth pulled and the end result being like, oh, that's why this is the outcome.
		Laura: age 55 [was the grand realization]... I found out age 55 is I got extreme TMD that year... I had to go find out why...
		Alice: initially when I was in, I didn't actually realize that it's doing damage to me. I didn't realize it until actually I finished my first treatment probably a year or two later
		Nick: I think it was definitely probably like the last three years or so [that I started regretting ERO]
		Jessica: But as far as, like, making the connection, I hadn't really done that before I started reading about it.
	Realization of Harm, Shedding the Veil	Jordan: the moment I realized that actually I did have premolar extraction was the day after my surgery because I was looking on forums and stuff like that on [redacted] and someone asked a photo of my palate, they said, oh, you have only twelve teeths, so I can see you had premolar extraction. I was like, oh, really? I didn't know that. I mean, I didn't even recall it because I'm 28 now. It was like 15 years ago almost
		Rick: He said, “Rick, your upper jaw is too far back. What happened to your upper jaw? I think I have an idea.
		Paula: It really wasn't until more recently getting ready for the jaw surgery that I started to understand that getting those teeth out was part of this whole issue
		Alice: And then when I think back the experience of my treatment, I actually remembered that, oh, actually halfway along the treatment, when they started retracting, I actually started getting the sign, but I actually didn't know...
		Nick: I kind of just coincidentally stumbled upon reddit posts or people talking about the negative consequences.
		Jessica: And I started reading a lot about what [redacted] was talking about with extraction retraction regret syndrome. And that was really when I started looking at my own history and thinking, like, wow, I've never really thought about this. But that actually lines up with my experience just because I thought about when I really started having a difficult time with my sleep. And it was really in my late teens, and my braces came off when I was 16, and I kind of just started thinking, like, I think that's me.
	Anger, Rage, Hate, Shame	Warren: Hatred is such a strong word, but I still want to use it. /Towards orthodontist in general?/ Yes. Or maybe not really? It's a weird combination of them doing this to me, but also not being able to get help.
		Jordan: But I had definitely had this regret you're talking about, this regret syndrome where I was very angry at my orthodontist. I actually went to see him last week to talk to him about it... I thought about how incompetent he was, but then I also rationalized and thought, well, it's the state of the vast majority of Orthodontics.
		Rick: Big time fucking rage. You couldn't believe. Murderous, murderous rage.
		Kalil: I had a lot of anger. I think I kind of told you, I was like, if I ever find that orthodontist, I'm going to file this huge class action thing that is just an anger reaction.
		Paula: it's been very frustrating and there are times where I'll kind of fall a little bit more into a cycle of kind of the like, wishing I wish things were different, I wish things were better, wish I could just find an answer
		Henrietta: just a little regret that I prefer not to have had that procedure done
		Laura: I feel ashamed that I don't have as much to offer other people. People used to think they enjoy being my company. I mean, there's all these leftover friends that when they see me once, now they don't want to see me again...
		Laura: I feel like [ERO} is a term that reminds me that we're in the Middle Ages when it comes to dental practices or orthodontic practices. And it makes me enraged because one of the problems about the disorder of premolar extraction syndrome is that it is not recognized or given any credence by the public... One of the worst things about having been mutilated with premolar extraction, because it is a mutilation, and I was mutilated. Okay, it’s that you cannot say it. You can say with Chinese footbinding. People say, oh, yeah, that was a mutilating social practice that went on for 1000 years. Which just shows what humanity is like. PR has only gone on for 80 years now, okay? Speaker 1 So hopefully it won't be 1000. Hopefully it'll be 100, tops and it'll be over with by the next generation. But it is a horrific experience to have another human being destroy your head, your skull and your physical ability to do basic human functions and nobody recognizes it... It's a taboo, and I know why it's a taboo, because if they really face the fact that they have spent their lives harming children, they rather prefer to deny it
		Alice: Why did I trusted her? Why did I not do more research? So you started blaming yourself.
		Nick: I would say regret, some sadness, some anger, but you can't really pin blame on any one person. It's just kind of like that's like the medical tradition. So that's just what people do. It's not like my dentist or orthodontist was out to get me or anything. That's just the way it's done.
		Jessica: So there's kind of that there's definitely a lot of anger. I just feel like even if I don't doubt that the Orthodontist had the best intentions and were following the guidance and all of the different stuff, but I really feel like they didn't help my airway problems at all. And that makes me quite angry.
		Nancy: Anger? Not so much. That's why I believe the dentist, because she looks very nice person. She made me believe. And even I have all this kind of problem. I go to see her, she's doing things so slowly but not means she has like a bad attitude. She's like she's she still look very nice... Yeah, I hate myself more.
	Depression, Sadness, Hopelessness, Regret	Kalil: following that was like a year or so of grief. I had this kind of idea that I was deficient somehow, and I just kind of carried that with me. In interactions, I'd go out and I'd feel like less than because I was like, oh, this happened
		Nancy: ... the memory is always like I keep like thinking, thinking why I finally take the dentist decision, the dentist suggestion. So it's like the regret killing me. So every day, sometimes I can’t stop crying, and have a nightmare.
		Rick: I was very depressed. Depression, but no, not depression. I was depressed, but didn't have depression. Depression is chronic... There were moments when I was like there was one moment, one moment when I was at that moment, I was in shock. I was like, oh, my God, it's 10:00 at night. I have to go to bed. I don't have time to Google treatment. I was very sad then. I wasn’t depressed. I was sad... And then I felt hopeless. But every time the headache came and I felt like killing myself, five minutes later, I was like, I'm going to beat this shit
		Laura: And this is where suicide comes in. When you feel that you're not productive, when you feel like you are not contributing anything except making people feel uncomfortable
		Alice: And then I was sad. I think I went into the grief stage. I was sad because I didn't know that it's reversible. I was not sure. So I just thought, oh, I've done something terrible to myself, and now there's no way back. And I was really sad that I'll be like this for the rest of my life. So really sad.
	Acceptance, Growth, Learning, Hope	Rick: "Beyond surviving. I’m thriving... Working a lot, traveling all over."
		Paula: it's kind of been a really fascinating journey and a really interesting learning experience
		Laura: The light thing is. In this situation, the few people who have shown empathy or come who have been good people, not only for me, the people I value good people.
		Alice: And then I and then I just didn't want to give up. I still just think thatI don't want to give up... I was thinking that if I can retract it back, I want to push it out and I want my teeth back.
		Nick: the more you learn about it yes, it's like it can be frustrating and depressing, but at the same time, being educated, it allows you to seek treatment. For me right now, yes, I'm frustrated, but the knowledge that one day I could get treatment and possibly have a reversal in some of these symptoms is pretty exciting
		Kalil: I was obsessively researching treatments, and I still always had hope, and I had this drive that I'm going to fix it and get to perfect health, and I still have that. I'm probably going to keep pursuing treatments after this, for sure. So despite all of it, it gave me kind of like a purpose. Like, okay, I'm sad, this sucks, but I'm going to find a way to fix it, and I'm going to share my journey along with this because this is kind of groundbreaking
Multifacetted Health Complaints	A full accounting of the MHC of each participant is listed in Table 2	
	Sleep breathing	Rick: He said, "Your airways are quarter the size it should be and it pulled your upper jaw back"
		Warren: when the spaces were close to about maybe one third... I started to experience really strange issues with my breathing and especially like I couldn't fit my tongue in my mouth... I started to snore like a locomotive...
		Henrietta: I'll have some days that I sleep pretty good and then I feel okay, and then there's some days that I don't sleep that great
		Laura: Suddenly I'm really always tired in the morning.
		Alice: very often that I wake up next morning feeling very lethargic, like very tired... I think maybe it's my tongue was telling you're, squashing me because I'm closing the space. You're squashing my room. I'm dying. I think it's my tongue telling me.
		Nick: I was getting concerned with my inability to breathe through my nose
		Jessica: I got my diagnosis of obstructive sleep apnea
		Jordan: I went to see a psychiatrist who actually told me most likely has sleep apnea because I look tired and I had retreating jaws
		Kalil: Sleep apnea is just one of them.
	Aesthetic	Rick: I felt ugly. Appearance also fucking butt ugly. I was always insecure about my jaw
		Paula: it contributed to longer narrow jaw and face and before the jaw surgery is much more like retracted lower jaw and upper jaw
		Alice: it made me look maybe 20 years older.
		Nick: My side profile looks a little strange
		Jessica: it's all contributing with this really long, narrow face and not a lot of definition and all that
		Nancy: After the three years I finished the whole treatment, I found out, it's hard for me to smile. When I smile, it's hard to show my teeth... When I go to friends, I take pictures that I look so weird on the picture. In a daily life, you see a local, maybe it's okay, but on the picture, so weird.
		Kalil: braces pushed my entire mid face back and is essentially the root cause of a lot of my issues
		Jordan: I was always a bit self conscious about my profile because I had receding jaws
	Pain Patterns	Rick: It's hard for me because my TMJS are fucked up... Fucking constant neck and back pain
		Warren: I also started to experience weird back pains and neck pains... the reason I'm so heavily medicated now is because my back literally snapped.
		Paula: I guess kind of my whole life I had a lot of head and neck pain
		Alice: When the TMJ is hurting, it actually will sort of like initially it was TMJ, and then it's here, and then it's headache, and then it gives migraine. It's one problem. It's one after another. And that pain. It doesn't go away. It just doesn't go away. And when that happens, it doesn't happen every day, all the time. No, but when it happens, I can't do anything that day because when you have migraine, I just have to lie down. I can't do anything. So it's affecting me work. It's affecting my social life. Like, when I'm talking to friends because I'm in pain, I tend to shorten my conversation. I tend to talk less, so I don't talk anymore. Oh, and then that affected my mental health, obviously.
		Jessica: I had had a lot of facial tension and pain that I wasn't really aware of
		Jordan: I think that I have a really nasty lower back pain that also significantly reduced my quality of life for the past three and a half years.
	Neuropsychobehavioural	Warren: I couldn't keep up with school, I could not concentrate
		Paula: I just remember kind of dragging myself up the stairs and just being like so exhausted and brain fog like you wouldn't believe
		Nick: And then I also have ADHD, which, again, could be caused by sleep apnea.
		Jessica: Anxiety and depression.
		Nancy: I cannot focus doing anything. Sometimes I really want to focus doing my job, but I cannot
		Jordan: ... I was always tired in high school and university. I would fall asleep in class and I would have trouble focusing... and ADHD
		Kalil: ADHD and lack of attention and other things that are associated with not getting enough oxygen to your brain at night
	Many Others	See Table 2
Finding solutions and coping strategies	A full accounting of interventions trialed is listed in Table 2	
	Difficulty finding competent healthcare providers	Warren: Getting help is one thing, but just to get an appointment to be able to talk about this can take forever... to wait that long to then just hear that, no, you're not going to get it. Just like you said, it's very disheartening.
		Paula: when I first started experiencing these symptoms, and I went to my general practitioner at the time, and they were kind of like, literally, this was my experience was that I saw a couple of different people who, when I would explain to them, like, my fatigue and my symptoms and they would sort of not investigate anything, but they would offer me antidepressants on the spot
		Laura: I probably saw about 350 people in eight countries... So when the first symptoms started happening, I saw maybe four doctors a week...
		Nick: it's a lot easier to get prescribed antidepressants than it is to get a sleep study at my age
		Jordan: Not diagnosed the problem, they treated symptoms
	Variable Effectiveness, Safety, Cost of Interventions	Rick: "Oral myofunctional did a little bit for me. Not a lot... I spent about 25 grand on consultations."
		Paula: I have the financial resources to do what I need to do... I think probably what has helped the most is doing some of these things consistently for the past ten years and just that slowly over time have given my body enough support to get a little bit better
		Laura: I did MSE. Naively with a young bright orthodontist in Germany who installed it wrong and made my face asymmetrical. My whole posture has become asymmetric... I have so much bone loss and gum damage.
		Nick: I think nose surgery, it was a pretty good result, but I still suffer from allergies, so I can't say I'm 100% satisfied with that yet
		Nancy: At the beginning, I don't want to eat any medicine. I think maybe I can improve my body condition by myself. Now I finally cannot. So I started taking the pills for sleep.
		Jordan: So the psychiatrist gave me antidepressants and Stimulants and psychotherapy. The ENT doctor cut my turbinates and he didn't even do submucosal resection. He just cut them off.
		Kalil: I did everything, like the Alf and mewing and myofunctional therapy and all that kind of stuff...
Wishing There Was a Better Way	Palatal expansion as an alternative	Kalil: I think anyone under 16, even that age, could benefit from palatal expansion
		Paula: my Orthodontist now even was saying, like, yeah, we really do this differently now, and really focus on the expansion and kind of make sure that we don't have to pull teeth
		Jordan: if we would have left the teeth and did palate expansion instead of extraction, I would never had the unattractive jaws and developed sleep apnea.
		Henrietta: I don't even know if they did expansion at that time.
	Myofunctional therapy as an alternative	Rick: They could make ends meet by practicing orthotropics. Myofunctional therapy.

ERO treatment course

In the ERO Treatment Course, There were “Various Motivations For Seeking Orthodontic Intervention”. Participants were either referred by their general dentist for a malocclusion or sought orthodontic providers proactively through an intrinsic motivation to treat an obvious malocclusion for aesthetic, functional, or social reasons. A few participants had no discernible malocclusion and sought orthodontic providers as a general checkup prophylactically or because orthodontic intervention was seen as a social norm. Some participants identified underlying risk factors that were left unaddressed that led to their malocclusion, “Unaddressed Orofacial Myofunctional or Parafunctional Etiology”, such as ankyloglossia and poor tongue posture, abnormal swallowing, mouth breathing spontaneously or secondary to allergic rhinitis, thumb sucking, a complicated birth, lack of breastfeeding, and/or hard rigorous chewing. Unanimously, it was the “Orthodontist Who Suggests ERO”, while being described as medically necessary, the only option due to severity of the condition, or no explanation given at all. Finally, the “Treatment Goals Are Variably Met”, with some patients describing their teeth as straightened while others had ongoing malocclusion and most mentioned poor facial aesthetic outcomes. Usually, during the treatment course, multifaceted health complaints arose, while the initial denial in the ocean of grief and trauma inhibits immediately creating a link between treatment and adverse effects and looking for solutions.

Lack of informed consent

All participants mentioned that there was a Lack of Informed Consent. Some participants who received the intervention as children mentioned they were “Too Young to Understand” and that there was “Parental Influence in Making Decisions”. Some participants described that there was a “Trust In Orthodontists to Make The Right Decision”, that ERO is the “The Societal Normal of Treatment”, that there was a “Lack of Information on Alternatives”, and that were was a “Lack of Information on Risks”.

Ocean of grief and trauma

An Ocean of Grief and Trauma was identified in our participants with different polarities of emotional states to describe their overall emotional experience with ERO. Some participants described a delayed or gradual realization of the harm caused by ERO, an initial “Denial of Harm, Clinging to Safety”, saying they’d forgotten they had had teeth pulled, had repressed the memory as a traumatic experience, and were living with health complaints. Eventually, participants had a “Realization of Harm, Peeling the Veil”, often precipitating following a triggering event such as a dental practitioner counting the number of teeth, continued education and learning in regards to health complaints and finding information and peer support online, or through a haphazard cognitive construction. Other participants developed health complaints during or immediately after ERO and immediately attributed the treatment to their condition. Most participants expressed an “Anger, Rage, Hate, Shame” directed towards different vectors of their suffering: their ERO provider, the orthodontic profession as a whole, the ERO intervention specifically, society, the healthcare system, or themselves. Most participants described a “Depression, Sadness, Hopelessness, Regret” regarding their situation, a state of rumination on the ERO event as a source of ongoing pain and a disbelief that anything may reverse the damage. Some participants described a positive that goes along with the negative, a light with the dark, a post-traumatic growth period of “Acceptance, Growth, Learning, Hope” in which they’ve learned from their experience and find fulfillment in sharing their journey while keeping optimistic in future interventions improving their health.

Multifaceted health complaints

Participants described a variety of Multifaceted Health Complaints that were attributed to their ERO intervention. Some participants described ERO as the sole contributing factor for their health complaints, while others described a multifactorial etiology of nonoptimal craniofacial development with ERO as a contributing factor. Aesthetically, participants complained of long, narrow, retruded faces. Functionally, breathing complaints were common, both awake and during sleep, nasally and with a lack of tongue space. Pain patterns in the jaw joints, head, neck, and back were common complaints, along with a forward head posture. Neuropsychobehavioural issues were common, including concentration deficit, variable mood, chronic fatigue, and negative social/emotional/financial/professional effects. There were various other health complaints listed in Table 2, some of which included hormonal dysregulation resulting in hypothyroidism and amenorrhea, miscarriages, vertigo, bruxism, tinnitus, nicotine and alcohol addiction, spinal spondylosis, and others.

Finding solutions and coping strategies

Participants described how they’ve spent a lot of time and effort in Finding Solutions and Coping Strategies while trying a variety of interventions they have sought to address their multifaceted health complaints. These interventions are detailed in Table 2. Generally, there was “Difficulty Finding Competent Providers”, with many consultations necessary before the underlying symptom etiology were correctly identified and addressed. Among the interventions sought to help, there were “Variable Effectiveness, Safety, Cost”.

Wishing there was a better way

Often, participants would describe alternative interventions they would have preferred instead of the ERO, “Wishing There Was a Better Way”. These included rapid palatal expansion, non-extraction treatment, oral myofunctional therapy, or no treatment at all.

## Discussion

The subjective experience of patients with regret or dissatisfaction over previous extraction-retraction orthodontic treatment is explored. Patients revealed the large negative impact that this treatment has had on their lives. Their narratives were ones of broken trust, damaged health, and journeys looking to treat and improve their condition. This study is largely unique as, to our knowledge, no phenomenological research has been done on patient experience with orthodontic treatment, and certainly none focusing on negative experience with extraction retraction. Previous studies examining patient satisfaction with orthodontic experience found that satisfaction is associated with met expectations in regard to aesthetics, function, and general well-being [25]. This is congruent with our study in that our patients had poorly met expectations, had negative outcomes, and were correspondingly highly dissatisfied with their results. A previous study found similar levels of satisfaction in extraction versus non-extraction patients after long-term follow-up [26], while our study focuses on dissatisfaction with extraction retraction orthodontic patients to prevent the occurrence of such.

The theme “ERO Treatment Course” encapsulates the narrative journey the participants embarked upon when they underwent extraction retraction orthodontic treatment. Samsonyanová and Broukal (2014) performed a systematic review of patient motivation for seeking orthodontic care and found that aesthetic reasons were the most common, with various social and functional reasons present as well [27]. This is congruent with our results. We found that there are often unaddressed contributors to the malocclusion. Factors influencing craniofacial development are an ongoing research topic [28]. We found that it is always the orthodontist who suggests ERO, never the patient. This is incongruent with the results of Klock (1995), who found that in a Norwegian population, it was the patients’ decision to extract in a third of cases, the dentists’ in two thirds, and that patients felt like they had an influence in the decision [29]. This may be indicative of poor patient-provider communication in our population, which Zadake (2020) found to be an essential factor in patient satisfaction with orthodontic treatment [30]. Despite the evidence supporting the effectiveness and safety of ERO in achieving optimal treatment outcomes [10,11,14-22], our patients had a variably met treatment goals, often with ongoing malocclusion different from the initial, poor facial aesthetics, and an overall decrease in their quality of life and health, representing a large incongruency in the available literature and an essential knowledge gap that requires additional investigation.

The theme, “Lack of Informed Consent”, was determined as most participants described an inability to actively choose the given intervention. For participants who underwent ERO as children, they attributed lack of informed consent to being too young to know what was going on and trusting adults to make the correct decision. Katz and Webb (2016) described pediatric informed consent as a combination of both parental permission and child assent, with assent including the developmentally appropriate understanding of the condition and treatment process, checking for understanding and influential factors, and soliciting an expression of willingness to participate [31]. Adewumi et al. (2001) found that when children gave verbal assent to dental treatment, they felt they were more involved in deciding to treat than those who did not, and concluded that children want to be more involved in consenting to their dental care [32]. Atsaidis et al. (2022) performed a systematic review on the effectiveness of the consent process on pediatric surgery and found effective consent included the use of multimedia, multiple conversations, and tailored communication, while ineffective strategies included poor parental understanding, too much information, or not addressing concerns [33]. This is consistent with our study as our patients felt like they were not involved in the consent process as children, and it seemed that poor communication strategies were utilized by their orthodontist. For the patients who underwent ERO as adults, hesitancy was noted upon their decision to move forward with the intervention, while they cited both lack of alternative treatment options given and description of the risks of the procedure. Improving and assessing the effectiveness of the informed consent process is an important and necessary research avenue in orthodontics.

The theme “Multifaceted Health Complaints” describes the variable detrimental health effects patients ascribe partly or entirely to their ERO treatment. The typical adverse effects of concern with orthodontic treatment include tooth pain, mucosal irritation, gum recession, root resorption, or temporomandibular joint (TMJ) [34]. Most of our patients complained of sleep breathing difficulty. Research on the effects of ERO on the airway is largely heterogeneous, low quality, or high risk of bias [18]. Aesthetically, most of our patients complained of craniofacial recession and a long, narrow face. The literature surrounding ERO on facial changes is also heterogeneous and low quality, with a high risk of bias, though there seems to be a pattern of retrusion similar to what our patients describe [19]. Our patients reported a variety of miscellaneous health complaints related to ERO that have not been well documented in the literature. These included musculoskeletal injuries, poor posture, pain patterns, poor exercise performance, vertigo, negative social, emotional, financial, and professional effects, miscarriages, hormonal effects, gastroesophageal reflux disease, attention deficit disorder, depression, anxiety, and addiction. Further investigation into the adverse effects of ERO is indicated.

The theme “Ocean of Grief and Trauma” describes the fluid emotional landscape of the participants. Our described ocean is similar to both Kübler-Ross’s five stages of grief and Walker’s complex post-traumatic stress disorder (CPTSD) four F typology [35,36]. Similarly to Ross’s stages, we found a pole for denial, anger, depression, and acceptance. These poles are all self-protective and serve various purposes for the individual with denial being a flight from reality, where the negative event, ERO, is repressed to minimize emotional pain, while anger is a more conscious and active rejection of reality, a fight response, protesting unfairness and bringing forth change, and depression a halting, recuperative, reflective freeze response where some of the pain that the uncomfortable reality holds may be felt. Eventually, through the grieving process of fully feeling the anger and sadness, an acceptance pole is realized, a post-traumatic growth state of peace, understanding, forward direction, with the possibility of even feeling gratitude and joy similar to a fawning response. Corr CA (2019) discusses the limitations of applying Kübler-Ross’s stages into practice and mentions the lack of distinct observable stages and how some authors emphasize the importance of shock, anxiety, and disbelief in the emotional process [37]. This is congruent in our study and is the reason an ocean is described rather than a cycle, in which participants actively flow between poles while displaying various elements of each, with variation in phenotype and degree of healthy calibration. Indeed, being in the ocean is an uncomfortable state, with being shocked at the frigid temperatures, turbulent waves, and fearing the depths. Notably, our process lacks a bargaining stage in which patients attempt to obtain a measure of control of their situation as this is encompassed in the theme “looking for solutions”, which likely has its own emotional landscape that is beyond the scope of this general overview study. Our acceptance pole differs from Ross’s in that it is a more active state of growth, learning, and moving forward rather than a lack of protest and struggle, likely due to the differing etiology being a negative experience rather than a terminal diagnosis.

It was found appropriate to couple the grieving process with the four F CPTSD typology due to the overlap in poles and traumatic fight/flight/freeze/fawn responses. Bracha and others (2006) describe the posttraumatic dental care anxiety (PDCA) as the most clinically accurate term for dental anxiety or specific dental phobias that arise from aversive dental experiences, while Hussein and others (2022) found that behavioral guidance techniques were effective in children with dental anxiety for reducing the need for general anesthesia or sedation for dental procedures [38,39]. Our study provides insight into the expression, connection, and progressive nature of dental anxiety, PDCA, CPTSD, and the grieving process and highlights the need for further research and care innovation for the prevention and treatment of such.

The theme “Wishing There Was a Better Way” was determined as participants described the treatments they wished they had received instead of ERO. Rapid palatal expansion and oral myofunctional therapy were mentioned as preferred alternatives and are congruent with published literature in that there is supportive evidence on the beneficial effects of rapid palatal expansion (RPE) and orofacial myofunctional therapy (OMT) on the airway parameters and craniofacial development, though further high-quality research is warranted [40-42]. Patano et al. (2023) performed a review on various potential orthodontic treatment options for crowding and mentioned arch perimeter increment, mesiodistal width reduction, or extractions, with the mandible being the most challenging aspect of arch expansion, and that roughly 3 mm of space may be recovered by distalization and vestibularization using various appliances like the Schwartz [43]. Gül et al. performed a survey on patients who underwent surgically assisted maxillomandibular transverse expansion and noted high satisfaction rates, suggesting a well-tolerated non-extraction therapy option [44]. 

This study has several strengths and limitations. The patient population was self-recruited and predominantly negatively biased towards ERO. This is both a strength and a weakness in that it provides valuable insight into dissatisfaction following ERO while representing a narrow spectrum of reality. The methodology is another strength and weakness in that phenomenology is unconcerned with proving causation or strength of correlations but seeks to explore subjective experience, allowing us to understand how patients experience the world and inform us on how to address their concerns. This study was not designed to explore the incidence, the entire breadth of possible adverse effects, or the best possible treatment of ERO patients. In IPA and double hermetic analysis, the resulting themes are as much a reflection of participant experiences as the investigator’s understanding of their experiences. As the investigator (JA) has lived experience with ERO, bracketing was used during data analysis to reduce the effect of personal bias, and fully anonymized and annotated transcripts are provided with supplementary material to increase trustworthiness.

## Conclusions

This study provides insight into the nature of procedural regret and dissatisfaction with extraction retraction orthodontic treatment. Participants discussed their experience with ERO, the health complications they believe resulted from their ERO treatment, the difficult emotional landscape their journey entails, and the interventions they’ve tried to improve their condition. Their procedural regret is centered around the health complaints that arose following the extraction retraction. Patients felt like they were not given the ability to provide good informed consent for this intervention. This study highlights several essential knowledge gaps in the literature and future research needs. To prevent regret following ERO, it is necessary to improve and assess the informed consent process and to identify and modify the factors affecting craniofacial growth while promoting non-extraction retraction therapies.

## Appendices

**Table 4:** Consolidated criteria for reporting qualitative studies (COREQ): 32-item checklist

No	Item	Guide questions/description
Domain 1: Research team and reflexivity		
Personal Characteristics		
1.	Interviewer/facilitator	Which author/s conducted the interview or focus group? JR conducted the interviews
2.	Credentials	What were the researcher's credentials? Bachelor of science in pharmacy, PharmD candidate
3.	Occupation	What was their occupation at the time of the study? Registered and practicing pharmacist
4.	Gender	Was the researcher male or female? Male
5.	Experience and training	What experience or training did the researcher have? Didactic qualitative research training at the undergraduate and graduate level, extensive reading and self-learning, mentorship by professionals with experience performing PhD dissertations in this methodology
Relationship with participants		
6.	Relationship established	Was a relationship established prior to study commencement? There was a prior relationship established with one participant, minimal relationship with three other participants (limited to corresponding via message boards or social media comments), and no relationship with the rest
7.	Participant knowledge of the interviewer	What did the participants know about the researcher? e.g. personal goals, reasons for doing the research Participants were made to understand the goal of the research project was to understand the experience of those who regret or are dissatisfied with previous extraction-retraction orthodontics
8.	Interviewer characteristics	What characteristics were reported about the interviewer/facilitator? e.g. Bias, assumptions, reasons and interests in the research topic Participants were made aware of the investigator’s own experience with extractive retractive orthodontics
Domain 2: study design		
Theoretical framework		
9.	Methodological orientation and Theory	What methodological orientation was stated to underpin the study? e.g. grounded theory, discourse analysis, ethnography, phenomenology, content analysis Interpretive phenomenological analysis, double hermetic thematic analysis
Participant selection		
10.	Sampling	How were participants selected? e.g. purposive, convenience, consecutive, snowball Convenience purposive sampling
11.	Method of approach	How were participants approached? e.g. face-to-face, telephone, mail, email Online message board or email
12.	Sample size	How many participants were in the study? 11
13.	Non-participation	How many people refused to participate or dropped out? Reasons? There were roughly 16 initial leads via email. Some never responded with availability for the interview. One did not respond with demographic questions after giving the interview and informed consent and their interview transcript was not included
Setting		
14.	Setting of data collection	Where was the data collected? e.g. home, clinic, workplace Audio recording of a video conference
15.	Presence of non-participants	Was anyone else present besides the participants and researchers? No
16.	Description of sample	What are the important characteristics of the sample? e.g. demographic data, date Described in Table 2
Data collection		
17.	Interview guide	Were questions, prompts, guides provided by the authors? Was it pilot tested? Semi-structured interviews were performed with pre-written question guides available in Table 1. These questions were pilot tested, reviewed and refined extensively, and based on previous similar studies
18.	Repeat interviews	Were repeat interviews carried out? If yes, how many? A single interview was found to be enough in all cases
19.	Audio/visual recording	Did the research use audio or visual recording to collect the data? Only audio recording was used
20.	Field notes	Were field notes made during and/or after the interview or focus group? No
21.	Duration	What was the duration of the interviews or focus group? Roughly between 30 minutes and an hour and a half
22.	Data saturation	Was data saturation discussed? It was believed interpatient thematic data saturation was reached after only roughly five participants. Recruitment continued to increase the confidence of thematic data saturation and increase the saturation of non thematic data such as variable patient characteristics, health complaints, interventions sought to help
23.	Transcripts returned	Were transcripts returned to participants for comment and/or correction? Yes, along with final manuscript draft before publication
Domain 3: analysis and findingsz		
Data analysis		
24.	Number of data coders	How many data coders coded the data? One (JR)
25.	Description of the coding tree	Did authors provide a description of the coding tree? Complete annotated transcripts are submitted for publication
26.	Derivation of themes	Were themes identified in advance or derived from the data? Derived from the data
27.	Software	What software, if applicable, was used to manage the data? Microsoft word
28.	Participant checking	Did participants provide feedback on the findings? Most chose to offer agreement before publication and expressed appreciativeness. Some chose to redact specific items from their transcript.
Reporting		
29.	Quotations presented	Were participant quotations presented to illustrate the themes / findings? Was each quotation identified? e.g. participant number Yes
30.	Data and findings consistent	Was there consistency between the data presented and the findings? Yes
31.	Clarity of major themes	Were major themes clearly presented in the findings? Yes
32.	Clarity of minor themes	Is there a description of diverse cases or discussion of minor themes? Yes
